# Spiral Jejunal Reconstruction of Both Pancreatic Stumps in Type II Portal Annular Pancreas during Pancreaticoduodenectomy: A Case Report

**DOI:** 10.70352/scrj.cr.26-0511

**Published:** 2026-10-31

**Authors:** Hiroto Suzuki, Ken Ishikawa, Takuya Bamba, Masataka Nakano, Yuki Inagaki, Kentaro Hamada, Sizuka Shoji, Mitsuo Mori

**Affiliations:** 1 Department of Surgery, Nagoya Tokushukai General Hospital, Kasugai, Aichi, Japan; 2 Department of Surgery, Shizuoka Tokushukai, General Hospital, Shizuoka, Shizuoka, Japan

**Keywords:** portal annular pancreas, spiral anastomosis, pancreatic resection, postoperative pancreatic fistula

## Abstract

**INTRODUCTION:**

Portal annular pancreas (PAP) is a rare congenital anomaly in which the uncinate process encircles the portal vein (PV) or superior mesenteric vein (SMV) and fuses with the dorsal aspect of the pancreatic body. We herein report a case of ampullary carcinoma associated with PAP treated by subtotal stomach-preserving pancreaticoduodenectomy.

**CASE PRESENTATION:**

A 51-year-old woman presented with scleral icterus and watery diarrhea. Following a detailed examination, she was diagnosed with carcinoma of the ampulla of Vater and underwent surgical treatment. During pancreatic resection, 2 pancreatic cut surfaces were identified on the ventral and dorsal sides of the SMV, leading to the intraoperative diagnosis of PAP. A lifted jejunal limb was rotated in a spiral fashion, and pancreaticojejunostomy was performed for each pancreatic stump. The postoperative course was uneventful, with no evidence of postoperative pancreatic fistula, and the patient was discharged in good condition.

**CONCLUSIONS:**

Although PAP is a rare anatomical anomaly, it requires careful attention during surgical procedures and reconstruction; therefore, it should be recognized preoperatively whenever possible. The reconstruction method employed in the present case may be useful as a reconstructive option for PAP because it avoids excessive pancreatic resection. We report this case with a review of the relevant literature.

## Abbreviations

CR-POPF
: clinically relevant postoperative pancreatic fistula
MPD
: main pancreatic duct
PAP
: portal annular pancreas
POPF
: postoperative pancreatic fistula
PV
: portal vein
SMV
: superior mesenteric vein
SSPPD
: subtotal stomach-preserving pancreaticoduodenectomy

## INTRODUCTION

PAP is a rare anatomical anomaly in which the uncinate process of the pancreas encircles the PV or the SMV and fuses with the dorsal aspect of the pancreatic body.^1)^ Its reported incidence ranges from 1.1% to 2.5%.^2,3)^ Clinically, this condition becomes significant during pancreatic resection, particularly with respect to determining the pancreatic resection line and selecting an appropriate method for pancreaticojejunostomy, thereby requiring careful consideration. The optimal reconstruction strategy for PAP remains controversial, particularly in type I and type II PAP, in which the MPD courses dorsal to the PV. We report a rare case of type II PAP treated with a spiral jejunal reconstruction technique and review the literature regarding its surgical management.

## CASE PRESENTATION

A 51-year-old woman with no past medical history was referred to our hospital with a chief complaint of scleral icterus and watery diarrhea. On physical examination, tenderness was noted in the central lower abdomen, but there were no signs of muscle guarding.

Blood tests revealed elevated hepatobiliary enzymes, including aspartate aminotransferase (AST) at 122 U/L, alanine aminotransferase (ALT) at 194 U/L, alkaline phosphatase (ALP) at 653 U/L, and γ-glutamyl transpeptidase (γ-GTP) at 124 U/L. Obstructive jaundice was evident, with a total bilirubin level of 10.0 mg/dL and a direct bilirubin level of 6.7 mg/dL. Tumor markers were markedly elevated, with carbohydrate antigen 19-9 (CA19-9) at 12960 U/mL, DUPAN-2 at 519 U/mL, and SPAN-1 antigen at 457.2 U/mL. No abnormalities in glucose tolerance were identified.

Contrast-enhanced CT demonstrated a 20 × 15 mm enhancing mass lesion at the ampulla of Vater, with upstream dilatation of the common bile duct, intrahepatic bile ducts, and MPD. No radiological evidence of lymph node metastasis or distant metastasis was observed. The MPD coursed dorsal to the PV, a characteristic finding of PAP. However, this anomaly was not recognized preoperatively, and the diagnosis was established only during surgery (**Fig. 1**). Endoscopic retrograde cholangiopancreatography (ERCP) revealed a type 2 tumor (Borrmann classification) at the ampulla of Vater causing biliary obstruction, for which an endoscopic retrograde biliary drainage (ERBD) tube was placed (**Fig. 2**). Histopathological examination of biopsy specimens confirmed adenocarcinoma. Based on these findings, the patient was diagnosed with carcinoma of the ampulla of Vater (cT2, cN0, cM0), corresponding to clinical Stage I according to the American Joint Committee on Cancer (8th edition). Following biliary decompression, surgical treatment was undertaken.

**Fig. 1:**
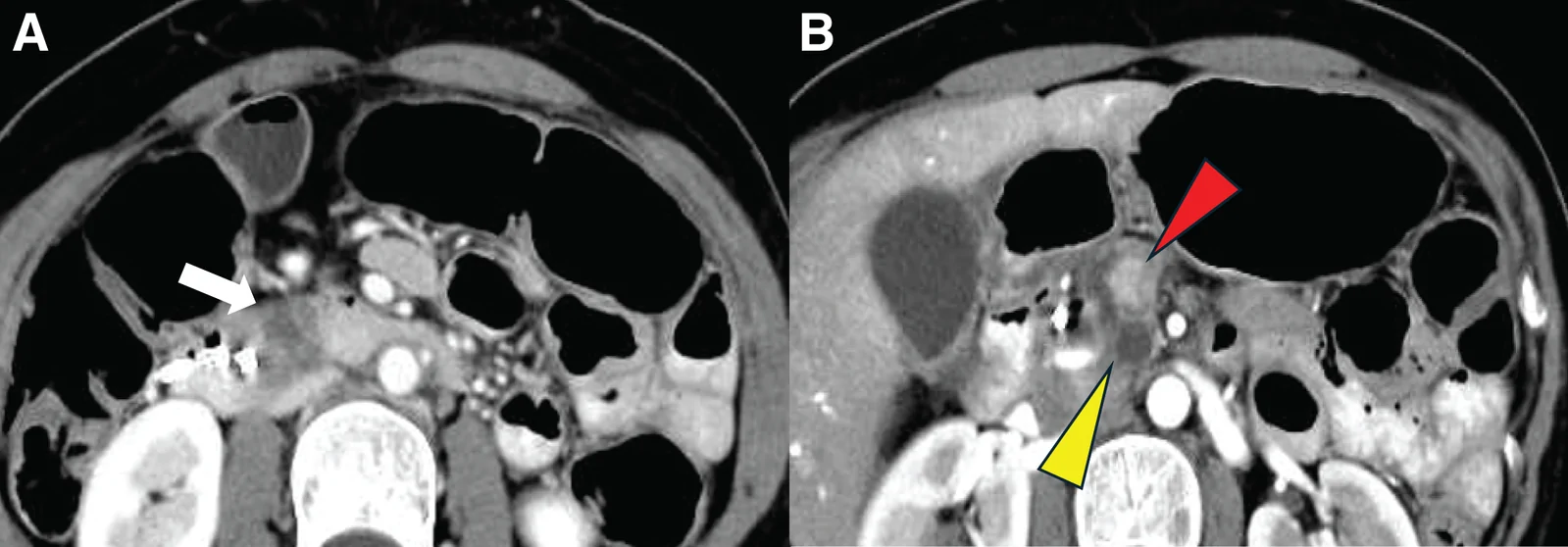
Preoperative CT findings. (**A**) The white arrow indicates a 20 × 15-mm enhancing mass lesion at the ampulla of Vater. No radiological evidence of lymph node metastasis or distant metastasis was observed. (**B**) The yellow arrow indicates the MPD, and the red arrow indicates the PV. The MPD coursed dorsal to the PV, and pancreatic parenchyma was also observed dorsal to the PV, findings consistent with PAP. MPD, main pancreatic duct; PAP, portal annular pancreas; PV, portal vein

**Fig. 2:**
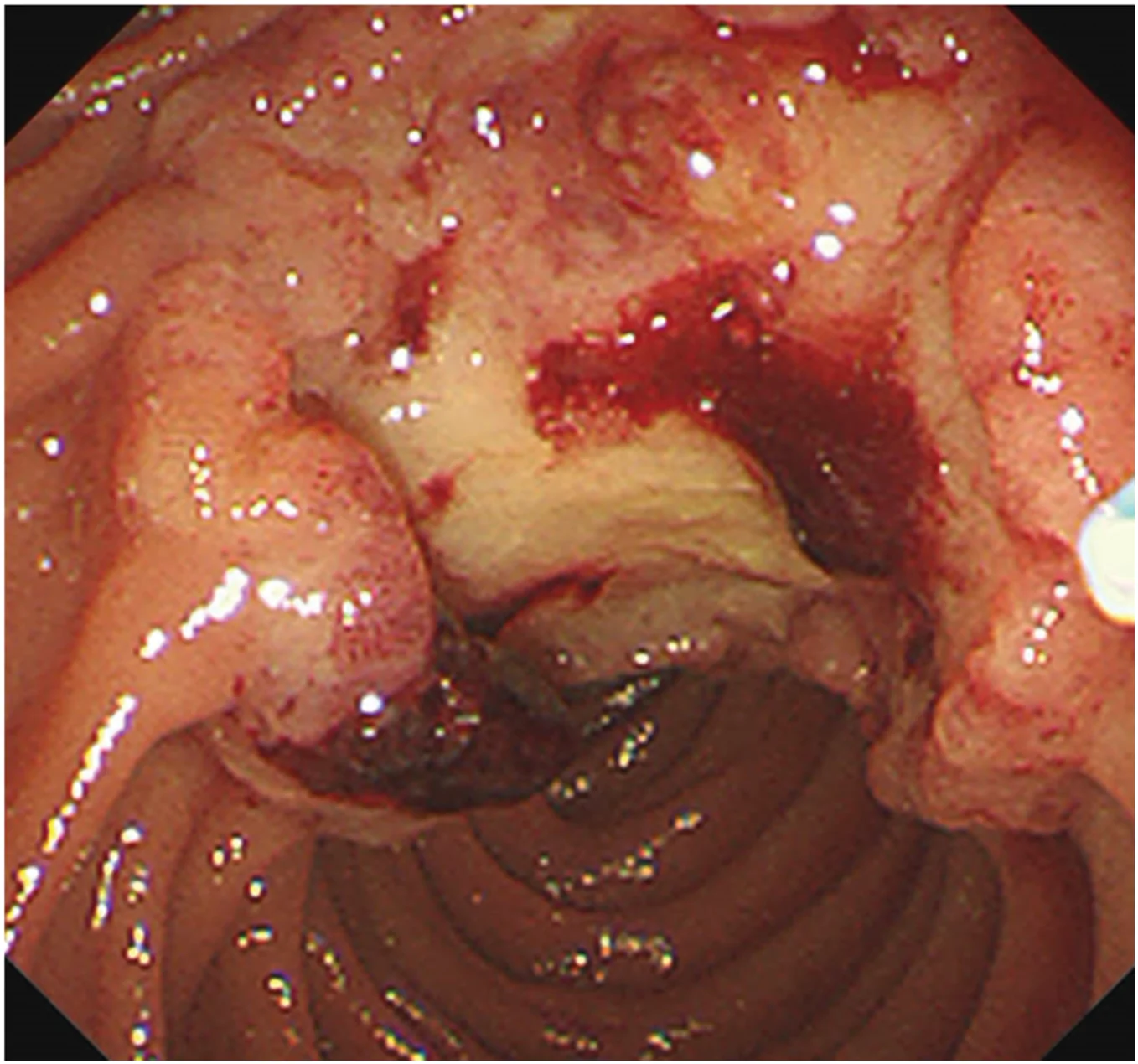
Endoscopic findings. A type 2 tumor was identified at the ampulla of Vater, causing biliary obstruction.

SSPPD with lymph node dissection was performed. PAP had not been identified preoperatively and was diagnosed intraoperatively. Through a midline upper abdominal incision, no ascites, peritoneal dissemination, or liver metastasis was observed. The pancreas was dissected and tunneled along the SMV and resected on its ventral aspect. Following pancreatic resection, during dissection of the pancreatic plexus from the dorsal aspect of the PV, pancreatic parenchyma was identified on the dorsal side of the SMV, extending continuously toward the left and fusing with the pancreatic body. A secretion of pancreatic juice was seen from the 2 respective pancreatic ducts on the 2 cut pancreatic surfaces. Based on these findings, PAP was diagnosed intraoperatively (**Fig. 3**). The dorsal pancreatic duct had a larger diameter and produced a greater amount of pancreatic juice than the ventral duct. Although PAP could not be diagnosed preoperatively, the preoperative CT images were re-reviewed intraoperatively, and the dorsal pancreatic duct was identified as the MPD. Because the MPD coursed dorsal to the SMV and the accessory pancreatic duct coursed ventral to the SMV, the anatomy was classified as type II PAP. As pancreatic juice was observed from both pancreatic cut surfaces, pancreaticojejunostomy was performed for each surface. The jejunum was elevated in a spiral fashion, followed by pancreaticojejunostomy to the dorsal pancreas, hepaticojejunostomy, and pancreaticojejunostomy to the ventral pancreas, in this order. To prevent twisting and redundancy of the elevated jejunal limb, the jejunum was brought up through the Treitz defect rather than through the transverse mesocolon, and the reconstruction was performed using the Whipple method rather than the Child method. The patient had a soft pancreas with a non-dilated pancreatic duct. A 5-Fr pancreatic duct tube could be inserted into the dorsal pancreas, where the MPD was located, but insertion into the ventral pancreas was difficult. Therefore, a 5-Fr lost stent was placed only in the dorsal pancreas. Both pancreatic anastomoses were performed using the invagination technique (**Figs. 4**–**6**). The total operative time was 540 minutes, and the intraoperative blood loss was 550 mL.

**Fig. 3:**
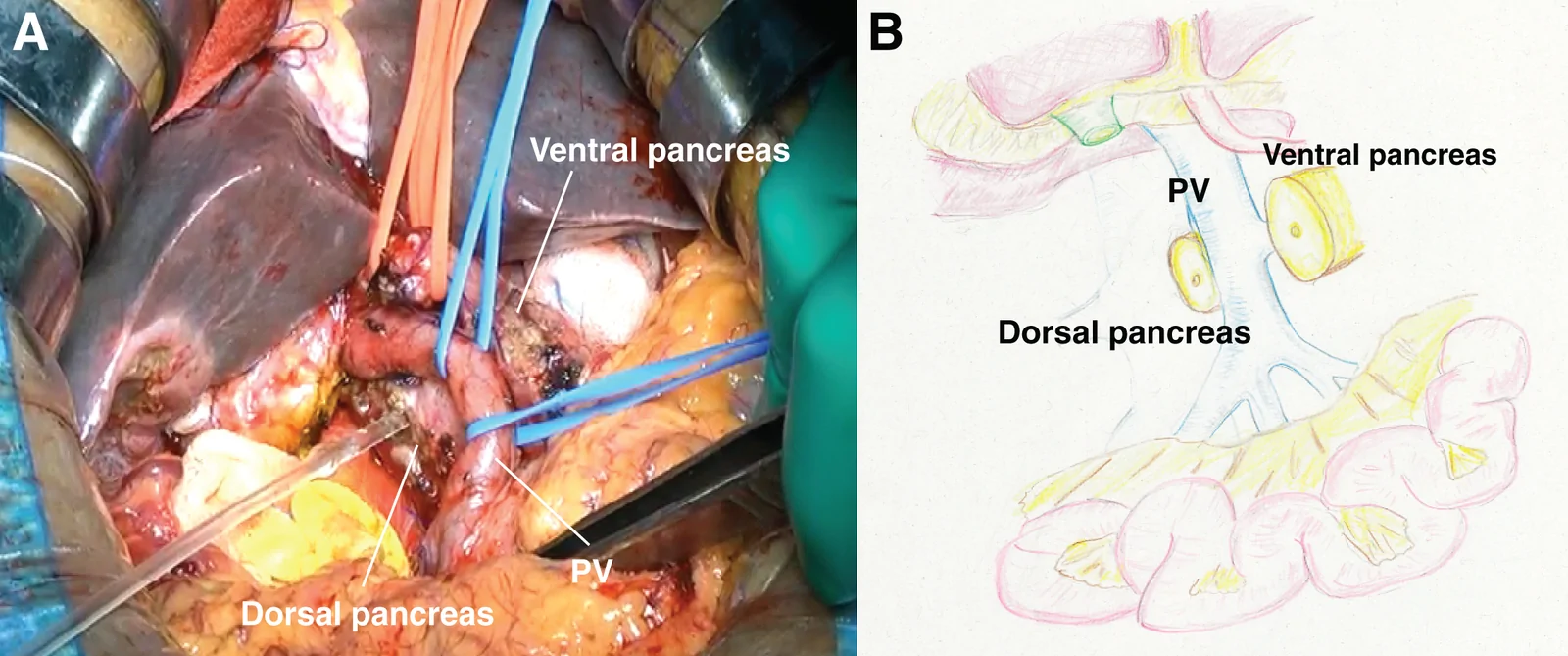
Intraoperative findings after pancreatic resection. (**A**, **B**) Following pancreatic resection, pancreatic parenchyma was identified on both the ventral and dorsal sides of the SMV. The MPD was found to course dorsal to the PV. MPD, main pancreatic duct; PV, portal vein; SMV, superior mesenteric vein

**Fig. 4:**
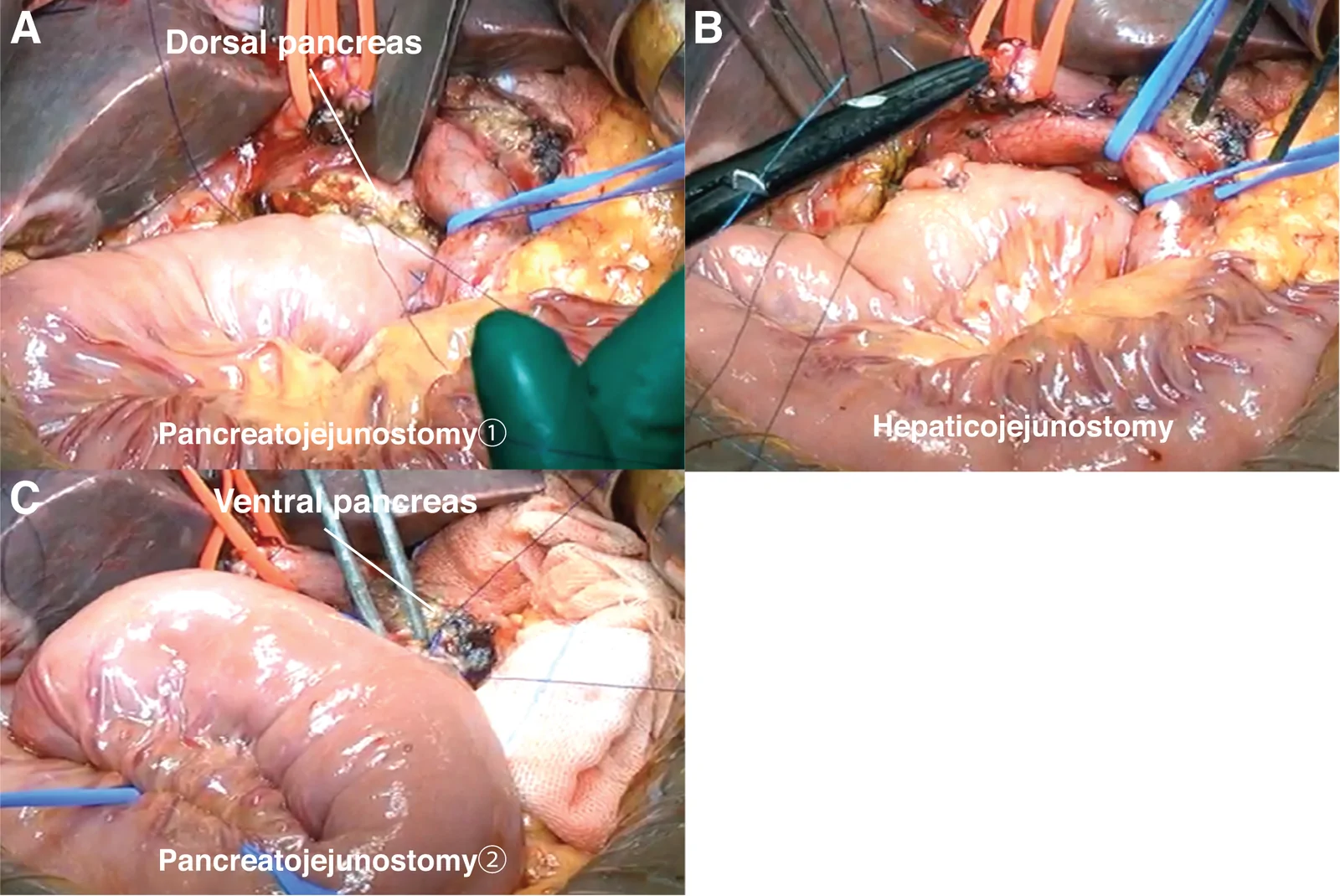
Spiral jejunal reconstruction for PAP. The elevated jejunal limb was brought up in a spiral fashion. Reconstruction was performed in the following sequence: pancreaticojejunostomy to the dorsal pancreatic stump (**A**), hepaticojejunostomy (**B**), and pancreaticojejunostomy to the ventral pancreatic stump (**C**). PAP, portal annular pancreas

**Fig. 5:**
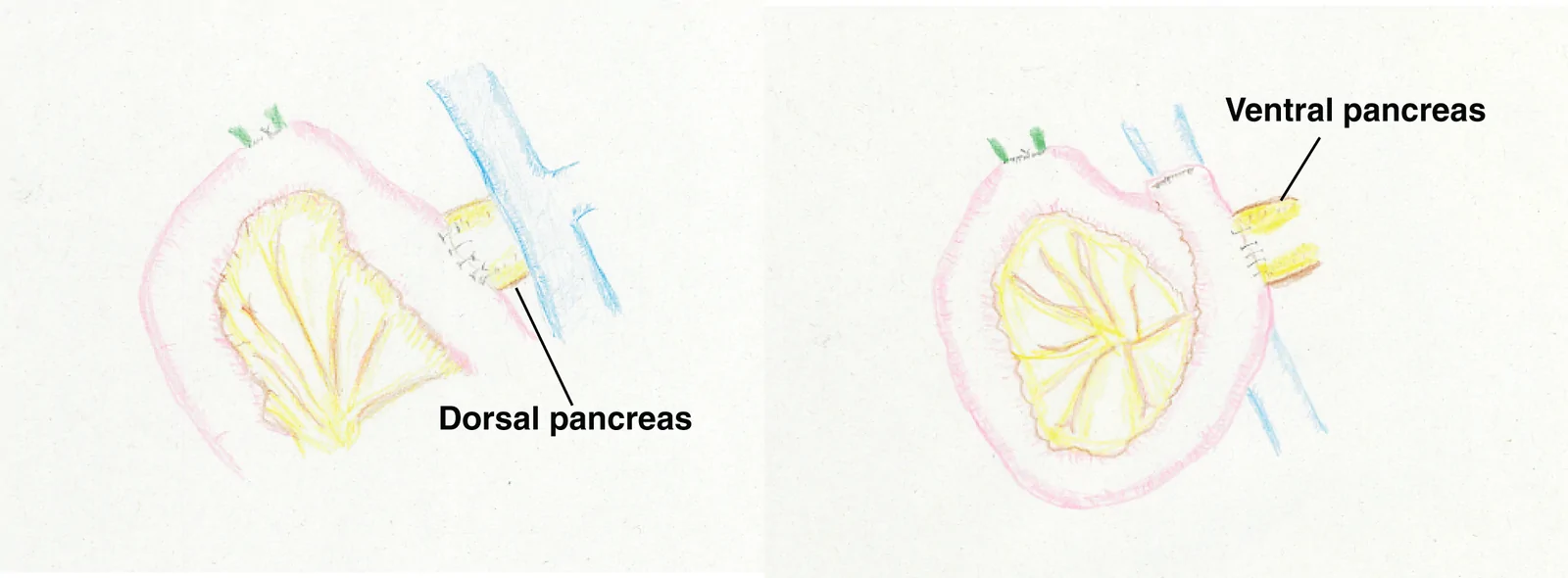
Schematic illustration of spiral jejunal reconstruction.

**Fig. 6:**
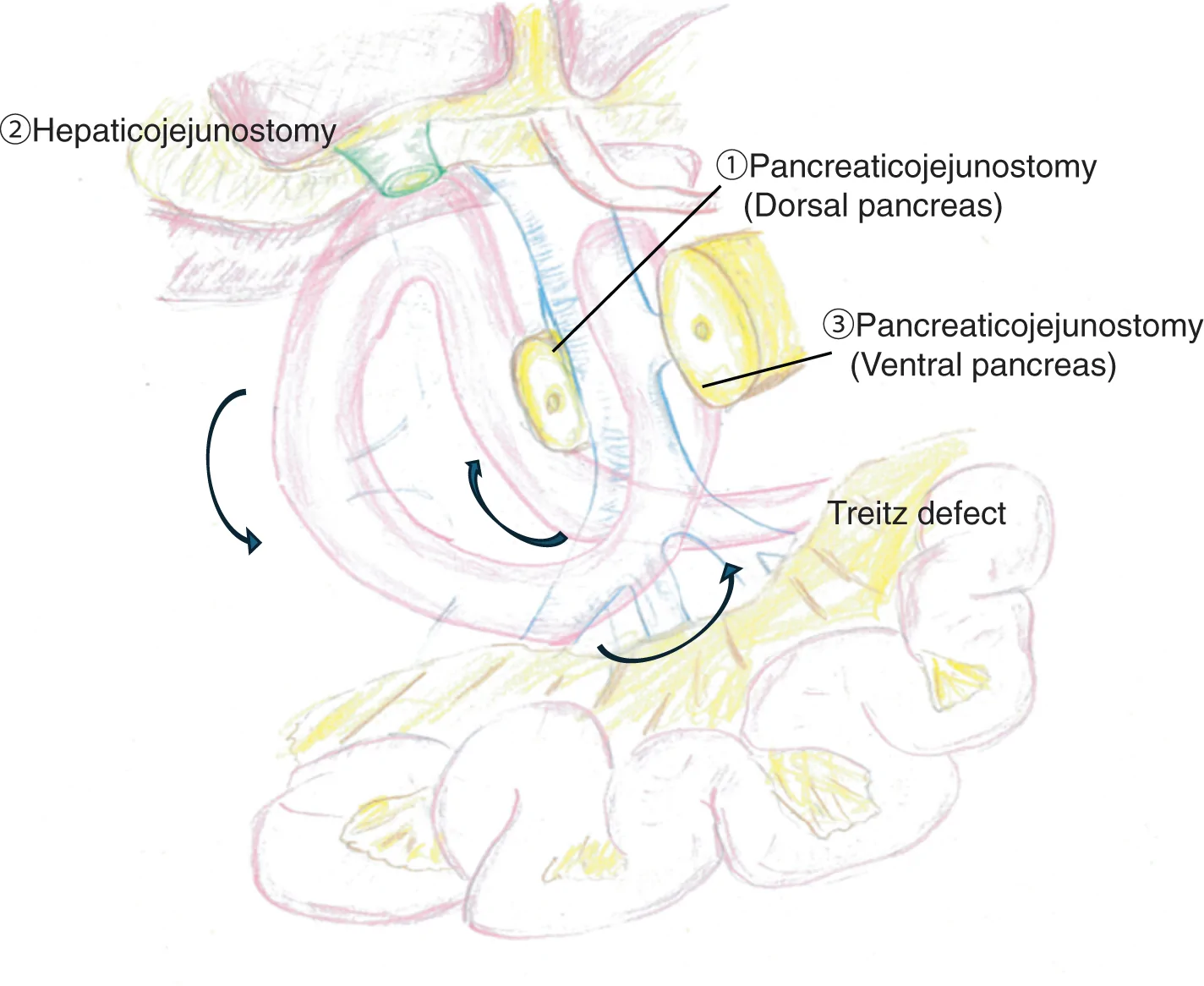
The course of the elevated jejunum.

Macroscopically, the pancreatic parenchyma demonstrated an annular configuration surrounding the PV. Histopathological examination confirmed adenocarcinoma of the ampulla of Vater, with a final pathological staging of pT3, pN0, pDM0, pHM0, pEM0, pPV0, pA0, and R0, corresponding to the pathological Stage IIA according to the American Joint Committee on Cancer (8th edition).

The postoperative course was uneventful, with no complications such as POPF. The patient was discharged on POD 17. Adjuvant chemotherapy with S-1 was administered for 6 months,^4)^ and the patient remains alive without recurrence approximately 20 months after surgery. No postoperative afferent loop syndrome or delayed gastric emptying occurred. Follow-up CT, performed every 6 months, has shown no evidence of pancreatic atrophy or pancreatic duct dilatation.

## DISCUSSION

The pancreas is embryologically formed by the fusion of the ventral and dorsal pancreatic buds at approximately 6–7 weeks of gestation. During this process, the uncinate process is considered to be primarily derived from the ventral pancreas, which rotates posteriorly from the dorsal side. PAP is defined as a rare morphological anomaly in which the uncinate process abnormally extends to the left during development and fuses in such a way that it completely encircles the PV.^1)^ Karasaki et al. reported an incidence of 8 cases (1.14%) among 700 randomly selected abdominal CT scans,^2)^ while Shimizu et al. identified 41 cases (2.05%) among 2000 patients without a history of pancreatic resection who underwent contrast-enhanced abdominal CT.^5)^

PAP has been classified by Joseph et al. according to the course of the MPD: type I, in which the MPD runs dorsal to the SMV; type II, in which the MPD and accessory pancreatic duct run ventral and dorsal to the SMV, respectively; and type III, in which the MPD runs ventral to the SMV. According to their report, type III is the most common (63%–97.5%), followed by type I (8%–22%), whereas type II is relatively rare (0%–14.8%).^6)^ The present case was classified as the less frequent type II.

A literature search of reports published between 1987 and 2025 was conducted using PubMed with the keywords “circumportal pancreas” and “portal annular pancreas.” A total of 63 cases undergoing pancreaticoduodenectomy (PD) for PAP were identified, making the present case the 64th.^1–3,6–42)^ Among 60 cases in which the Joseph classification was described, type II accounted for only 6 cases (10%), confirming its rarity, while type III and type I accounted for 48 cases (80%) and 6 cases (10%), respectively. The mean age was 67.7 years (range: 42–84); there were 34 male patients and 29 female patients, and cholangiocarcinoma was the most common diagnosis, accounting for 20 cases.

Although PAP is usually asymptomatic, it becomes clinically significant when pancreatic resection is required for concomitant disease. In such cases, careful consideration must be given to the determination of the pancreatic resection line and the method of reconstruction.^5)^ In standard PD, when an adequate distance from the tumor is ensured, the pancreatic resection line is typically set directly above the PV or along its left margin. However, in PAP, resection at this level necessitates division of the pancreatic parenchyma dorsal to the PV, resulting in 2 cut pancreatic surfaces. When 2 resection surfaces are present, the conventional approach is to ligate or close the surface without the MPD using a stapler, while reconstruction is performed only for the surface containing the MPD. In type III cases, in which the MPD runs ventral to the PV, the dorsal pancreas can be closed by ligation or stapling, and reconstruction can be performed on the ventral pancreas anterior to the PV. In contrast, in type I and II cases, in which the MPD runs dorsal to the PV, pancreatic resection near the PV often makes reconstruction technically challenging. In all scenarios, there remains concern regarding the risk of POPF arising from the non-reconstructed pancreatic stump.^2,24)^

PD for PAP has been reported to carry a high risk of POPF.^5)^ In our review, POPF was reported in 24 of 58 cases (41%) for which relevant data were available. After excluding biochemical leaks according to the International Study Group of Pancreatic Surgery definition, clinically relevant POPF (CR-POPF) occurred in 18 patients (31%). In recent years, several reports have described surgical techniques aimed at reducing the risk of POPF by setting the pancreatic resection line distal to the fusion site, thereby creating a single cut surface.^24)^ Among 56 cases with available data on the number of resection surfaces, CR-POPF occurred in 14 of 41 cases (34%) managed with 2 cut surfaces, compared with 4 of 15 cases (27%) managed with a single cut surface. However, the potential impact of excessive pancreatic resection resulting from limiting the pancreatic resection to a single surface should also be considered. Previous reports have suggested an increased risk of postoperative diabetes associated with a reduced remnant pancreas, as well as long-term atrophy of the remnant pancreas following PD.^5,28)^ Therefore, excessive pancreatic resection may lead to endocrine dysfunction. In the present review, when only type II cases were analyzed, 6 of 7 cases (86%) were managed with a single resection surface (**Table 1**).^6,12,14,17,25)^ This tendency may reflect the increased technical difficulty of reconstruction and the higher risk of POPF associated with the dorsal location of the MPD. In addition to the present case, Joseph et al. reported a type II PAP managed with 2 cut surfaces.^6)^ In their case, the pancreatic ducts at each cut surface were first ligated and sutured, followed by a side-to-side pancreaticojejunostomy. They explained that, in type II PAP, both the accessory pancreatic duct anterior to the PV and the MPD posterior to the PV need to be drained into the intestine.

**Table 1:** Summary of cases of PD in patients with type II PAP

Case	Year	Author	Age	Sex	Diagnosis	PAP type	Number of pancreatic cut surface	CR-POPF	Preoperative detectiom of PAP
1	2010	Joseph^6)^	51	M	Ampullary cancer	II	2	No	No
2	2012	Muto^12)^	45	F	Insulinoma	II	1	No	No
3	2012	Maejima^14)^	79	F	Bile duct cancer	II	1	N/D	No
4	2013	Matsumoto^17)^	81	F	Ampullary cancer	II	1	No	Yes
5	2017	Luu^25)^	81	M	Ampullary cancer	II	1	No	No
6	2017		60	M	Chronic pancreatitis	II	1	No	No
7		Our case	51	F	Ampullary cancer	II	2	No	No

CR-POPF, clinically relevant postoperative pancreatic fistula; F, female; M, male; N/D, not detected; PAP, portal annular pancreas; PD, pancreaticoduodenectomy

We therefore reviewed the reconstructive techniques used in cases requiring 2 pancreatic resection surfaces. For type III PAP, closure of the dorsal pancreas using a stapler or ligation, followed by reconstruction of the ventral pancreas anterior to the PV, is generally performed. In contrast, 4 previously reported cases of type I or type II PAP were managed using 4 different reconstructive techniques (**Table 2**).^6,8,20,29)^ This variation in surgical approaches may reflect the technical difficulty of performing pancreaticojejunostomy posterior to the PV, where the MPD is located. In reports by Narita et al and Kawamoto et al., the dorsal pancreas was dissected from the PV and mobilized to the anterior side of the PV, after which pancreaticojejunostomy was performed.^20,29)^ This approach is an effective option when reconstruction of the dorsal pancreas is required. In the present case, we did not consider this technique as an intraoperative reconstructive option because of insufficient recognition of PAP. However, this technique should be considered in patients with duodenal or ampullary carcinoma without tumor invasion or severe inflammation around the PV, in whom the dorsal pancreas can be safely dissected and mobilized from the PV. On the other hand, dissection and mobilization of the dorsal pancreas from the PV may be difficult when the pancreas is firmly adherent to the PV. In such cases, side-to-side pancreaticojejunostomy, as reported by Joseph et al., or pancreatic transection to the left of the PV may be considered.^6,8)^ The spiral elevation of the jejunum used in the present case provides another option when anterior reconstruction of the dorsal pancreas is difficult. This technique avoids dissection between the pancreas and PV and may reduce secondary pancreatic injury. It may also help avoid excessive pancreatic resection and preserve postoperative endocrine function.

**Table 2:** Summary of cases of PD with reconstruction at 2 cut surfaces in patients with type I or II PAP

Case	Year	Author	Age	Sex	Diagnosis	PAP type	Cutting line	Reconstruction	CR-POPF
1	2001	Mizuma^8)^	64	M	Gallbladder cancer	I	Left of the PV	Reduction of stumps using mattress suture→Both stump P-J	Yes
2	2010	Joseph^6)^	51	M	Ampullary cancer	II	PV	Both stump sutured→side to side P-J	No
3	2016	Narita^20)^	72	F	Pancreatic cancer	I	PV	Mobilization→Both stump P-G	N/D
4	2017	Kawamoto^29)^	76	F	Pancreatic cancer	I	PV	Mobilization→Both stump P-J	No
5		Our case	51	F	Ampullary cancer	II	PV	Spiral elevation of the jejunum →Both stump P-J	No

CR-POPF, clinically relevant postoperative pancreatic fistula; F, female; M, male; N/D, not detected; PAP, portal annular pancreas; PD, pancreaticoduodenectomy; P-G, pancreaticogastrostomy; P-J, pancreaticojejunostomy; PV, portal vein

The main limitation of this technique is that pancreatic resection at the PV line must provide an adequate oncological margin. Therefore, its use is limited to carefully selected patients. In the present case, the PAP was classified as type II. Pancreatic juice leakage was observed from the accessory pancreatic duct of the ventral pancreas. We therefore considered the risk of POPF to be high and performed pancreaticojejunostomy for both pancreatic resection surfaces. Although this approach increased the operative time, separate reconstruction of both pancreatic surfaces may not always be necessary. In selected type I cases, ligation of the ventral pancreas followed by fixation to the jejunum may be sufficient to prevent pancreatic juice leakage, as reported by Narita et al.^20)^ To our knowledge, no previous study has reported spiral elevation of the jejunum for reconstruction of PAP. Although this technique is relatively complex, it may be a useful option because it allows preservation of pancreatic parenchyma without extensive dissection around the PV. Reconstruction of the dorsal pancreas is technically demanding because of the limited operative space. When PAP is identified preoperatively, resection close to the right lateral wall of the PV may facilitate pancreaticojejunostomy. The invagination technique may help shorten the operative time when pancreaticojejunostomy is required for both pancreatic resection surfaces. In addition, the accessory pancreatic duct may be small in diameter, making duct-to-mucosa pancreaticojejunostomy technically difficult. In such cases, the invagination technique may be a useful alternative. In addition, bringing the jejunum through the Treitz defect may help prevent twisting and redundancy of the jejunal limb. In the present case, the postoperative course was uneventful, with no pancreatic fistula or endocrine dysfunction.

Although PAP is a rare anatomical anomaly, it can be diagnosed preoperatively using imaging studies. However, previous reports indicate that the rate of preoperative diagnosis remains low, which may be attributed to a lack of awareness of this condition or misinterpretation of PAP as peripancreatic lymph nodes or inflammatory changes.^2,23)^ Among the 64 cases reviewed, 40 had available data on preoperative diagnosis, and only 18 cases (45%) were correctly diagnosed before surgery. In our case, the condition was not recognized preoperatively due to insufficient awareness and was diagnosed intraoperatively. Preoperative consideration of the possibility of PAP and careful planning of the resection line and reconstruction strategy are essential for performing safe surgery without complications.

## CONCLUSIONS

We experienced a case in which PAP was diagnosed intraoperatively during PD. Although this condition is rare, it represents a risk factor for POPF; therefore, careful preoperative imaging evaluation with this anomaly in mind is essential. The reconstruction method employed at our institution may be useful as an option for PAP, as it enables avoidance of excessive pancreatic resection.

## DECLARATIONS

### Availability of data and materials

All the data and materials used in this study were obtained from publicly available sources or databases, and all cited literature is accessible through PubMed.
